# The effect of gastric fundus radiation dose on postoperative anastomotic leakage in esophageal cancer

**DOI:** 10.3389/fonc.2023.1080089

**Published:** 2023-02-28

**Authors:** Yulia Kundel, Noga Kurman, Omri Sulimani, Shlomo Gavrielli, Yuval Nachalon, Assaf Moore, Hanoch Kashtan, Eyal Fenig, Baruch Brenner, Aron Popovtzer, Elisha Fredman

**Affiliations:** ^1^ Department of Radiation Oncology, Davidoff Cancer Center, Rabin Medical Center, Petach Tikva, Israel; ^2^ Tel Aviv School of Medicine, Tel Aviv University, Tel Aviv, Israel; ^3^ Department of Surgery, Rabin Medical Center, Petach Tikva, Israel; ^4^ Department of Radiology, Rabin Medical Center, Petach Tikva, Israel; ^5^ Department of Otolaryngology – Head and Neck Surgery, Rabin Medical Center, Petach Tikva, Israel

**Keywords:** esophageal cancer, gastric fundus, radiation dose, postoperative, leakage, esophagectomy

## Abstract

**Introduction:**

Standard-of-care treatment for locally advanced esophageal carcinoma (LAEC) includes neoadjuvant chemoradiotherapy followed by esophagectomy. A potentially catastrophic surgical complication is the development of a postoperative anastomotic leak. To date, the association with radiation dose exposure had been inconclusive. We examined the correlation between radiation exposure to the gastric fundus and risk of postoperative leakage using contemporary radiation doses and fractionation.

**Methods:**

A total of 69 consecutive patients with LAEC who underwent neoadjuvant chemoradiotherapy followed by esophagectomy in our tertiary center were prospectively followed (median, 27 months). Neoadjuvant regimen included 50.4 Gy in 28 fractions with 5-fluorouracil and cisplatin and 41.4 Gy in 23 fractions with carboplatin and paclitaxel. The gastric fundus was contoured and dosimetric and radiation technique parameters were retrospectively evaluated.

**Results:**

Of the total number of patients, 71% and 29% had esophageal and gastroesophageal junction (GEJ) tumors, respectively. Fourteen patients (20.3%) experienced anastomotic leaks within a median of 2 days postoperatively, 78.6% of whom had lower third esophagus or GEJ primaries. Mean and minimum fundus dose did not significantly differ between those with and those without leakage (*p* = 0.42, *p* = 0.51). Mean fundus V25, V30, and V35 doses were numerically but not statistically higher in those with anastomotic leak (*p* = 0.58, *p* = 0.39, and *p* = 0.30, respectively). No correlation with incidence of leakage was seen between 3D and IMRT treatment modalities.

**Conclusions:**

In our comparatively large prospectively collected series of patients treated for LAEC, radiation dose to the gastric fundus during neoadjuvant combination therapy prior to surgery did not correlate with the risk of postoperative anastomotic leak.

## Introduction

The current standard-of-care treatment approach for the definitive management of locally advanced stage II and III carcinoma of the esophagus and gastro-esophageal junction (GEJ) is a multimodality approach consisting of neoadjuvant chemoradiation (nCRT) followed by surgical resection (1, 2). It has been suggested, however, that the addition of radiation to neoadjuvant chemotherapy may be associated with increased postoperative morbidity, and even mortality (3–6). One of the major and potentially catastrophic complications after surgery in this population is leakage of the esophagogastrostomy (EG) anastomosis (7), with rates in the literature ranging between 6% and 41% (1, 4, 8).

The question has arisen as to whether the degree of radiation exposure to the future EG anastomosis region, namely, the gastric fundus, correlates with and contributes to an increased risk of postoperative anastomotic leak. Thus far, reports have been inconsistent, with experiences suggesting an increased risk of leak when the anastomosis is placed within the irradiated field (9) and a correlation of increased dose with risk of a leak (10), while others failed to have a significant association (11). This is particularly relevant in the setting of a high prevalence of the subtype of esophageal tumors occurring in the distal third of the esophageal and GEJ (12).

In light of the ongoing uncertainty, this study aimed to further investigate the potential correlation, in the era of contemporary neoadjuvant radiation dose to the gastric fundus, as part of nCRT, with postoperative anastomotic leakage, in a comparatively large cohort of definitively treated patients.

## Materials and methods

### Patients

This prospectively collected cohort study included 69 consecutive patients with carcinoma of the esophagus and GEJ who were treated at our tertiary medical center through 2018. Clinical staging included endoscopic ultrasound as well as FDG PET-CT. All patients received nCRT, followed by three-field subtotal esophagectomy and reconstruction *via* gastric pull-up and cervical anastomosis.

### Treatment protocol

Patients treated earlier in the study period received standard-of-care nCRT composed of 50.4 Gy in 28 fractions with concurrent 5-fluorouracil (5FU) and cisplatin, later updated to 41.4 Gy in 23 fractions with weekly carboplatin and taxol as the CROSS protocol was adopted as departmental practice (13, 14). All patients underwent a contrast-enhanced CT simulation with arms raised and using appropriate immobilization. Target-volume contouring was performed based on rigid fusion of the diagnostic FDG PET-CT imaging, as per accepted standard practices (15) as follows. The gross tumor volume (GTV) was defined as the visualized primary tumor and clinically/pathologically involved regional lymph nodes based on imaging and endoscopy. The clinical target volume (CTV) was derived by a 3-cm craniocaudal and anatomically limited 0.5- to 1-cm radial expansion on the GTV with inclusion of the entire associated nodal compartments at those levels. Finally, an additional 1-cm planning target volume (PTV) margin was applied.

Radiation planning was performed on the Eclipse Treatment Planning System 16.1 (Varian Medical Systems, Palo Alto, CA), the initial 24 patients undergoing 3D-conformal radiotherapy (3D-CRT), and the final 45 receiving intensity-modulated radiotherapy (IMRT), utilizing 6-MV and 10-MV photons. Following a restaging FDG PET-CT, transthoracic esophagectomies were performed between 5 and 8 weeks after the completion of nCRT, with en bloc three-field lymphadenectomy and gastric conduit reconstruction.

### Outcomes

Patients were re-evaluated daily until hospital discharge following surgery, bi-weekly for 1 month, then every 3 months over the next 2 years. Anastomotic leakage was identified based on post-procedure clinical monitoring and postoperative radiography, with findings including demonstration of saliva through the cervical wound, extravasation of water-soluble contrast during a contrast swallow study or CT scan, or visualization of anastomotic dehiscence or fistulae during endoscopy or surgical reintervention.

In all cases, the gastric fundus was prospectively contoured on the pre-treatment simulation CT scan, first by an expert thoracoabdominal radiologist, which was then reviewed and confirmed by a dedicated gastrointestinal radiation oncologist. Any adjustments were made by consensus decision.

The following dose-volume histogram variables were calculated for the contoured fundus: mean dose, minimum dose, and dose that covered at least 50% of the volume, maximum dose, V25, V30, and V35 (i.e., percentage of the volume that received at least, 25, 30, and 35 Gy, respectively).

### Statistical analysis

Two-directional group comparisons for significance were performed using Student’s *t*-test and ANOVA. Univariate Cox regression for potential associations with leak parameters, including baseline patient and tumor characteristics, radiation delivery, and fundus dose parameters, was performed. Receiver operating characteristics analysis was performed for radiation dose parameters to identify ideal cutoff values in which equal weight was given to sensitivity and specificity. A *p*-value of less than 0.05 was considered statistically significant.

## Results

Of the 69 patients, 49 (71%) and 20 (29%) had esophageal and GEJ tumors, respectively. Patient and tumor characteristics were balanced between groups (**Table 1**). All patients had a pre-treatment Eastern Cooperative Oncology Group performance status of 0 (42%) or 1 (58%), and all patients completed their entire nCRT course.

**Table 1:** Baseline patient characteristics.

	No leak (*n* = 55)	Leak (*n* = 14)	T-test (*P-*value)
Female	38 (70%)	7 (50%)	0.15
Age (years)	68 ± 9	61 ± 10	0.17
ECOG			
0 1	24 (44%) 31 (56%)	6 (43%) 8 (57%)	0.85
Clinical TNM stage			
II (T2N0-1) II (T3N0) III (T3N1)	3 (5%) 23 (42%) 29 (53%)	1 (7%) 6 (43%) 7 (50%)	0.60
Adenocarcinoma histology	32 (59%)	9 (64%)	0.70
Tumor location			
Upper third	2 (3.6%)	1 (7.1%)	0.95
Middle third	8 (14.5%)	2 (14.3%)	
Lower third	29 (52.7%)	7 (50.0%)	
GEJ	16 (29.1%)	4 (28.6%)	
Radiation method			
3D	21 (38%)	3 (21%)	0.66
IMRT	34 (62%)	11 (79%)	
Total radiation dose			
41.4 Gy (23×1.8 Gy)	20 (36%)	5 (36%)	0.58
50.4 Gy (28×1.8 Gy)	35 (64%)	9 (64.5%)	

GEJ, gastro-esophageal junction; IMRT, intensity modulated radiation therapy.

Median follow-up after surgery was 27 months, with a median delay from completion of nCRT to surgery of 7 weeks. Postoperative gastric fundus anastomotic leak was experienced by 20.3% (14/69) of patients, 78.6% of whom had tumors located either at the GEJ or lower third of the esophagus. Median time to diagnosis of anastomotic leak was 2 days.

Mean and minimum dose to the gastric fundus between those with and those without an anastomotic leak were 35.7 Gy *vs*. 31.2 Gy (*p* = 0.42) and 13.7 Gy *vs*. 11.4 Gy (*p* = 0.51), respectively (**Table 2**). Mean fundus dose correlated significantly only with primary tumor location: 37 Gy for GEJ and lower third tumors *vs*. 9 Gy for upper and middle third tumors (*p* = 0.05). On univariate analysis, there were no patient, tumor, treatment, or dose factors significantly associated with anastomotic leaks, including radiation delivery technique.

**Table 2:** Gastric fundus radiation dose parameter comparison between those without and those with anastomotic leak.

Parameter	No leak (*n* = 55)	Leak (*n* = 14)	*t*-test (*p-*value)
Mean fundus dose (Gy)	312. ± 14	35.7 ± 14	0.42
Minimum fundus dose (Gy)	11.4 ± 10	13.7 ± 7	0.51
Maximum fundus dose (Gy)	36 ± 5	39 ± 3	0.54
V25 fundus (%)	65.6 ± 34	71.5 ± 34	0.58
V30 fundus (%)	55.6 ± 33	64.3 ± 33	0.39
V35 fundus (%)	48.4 ± 31	58.5 ± 33	0.30

**Figure 1:**
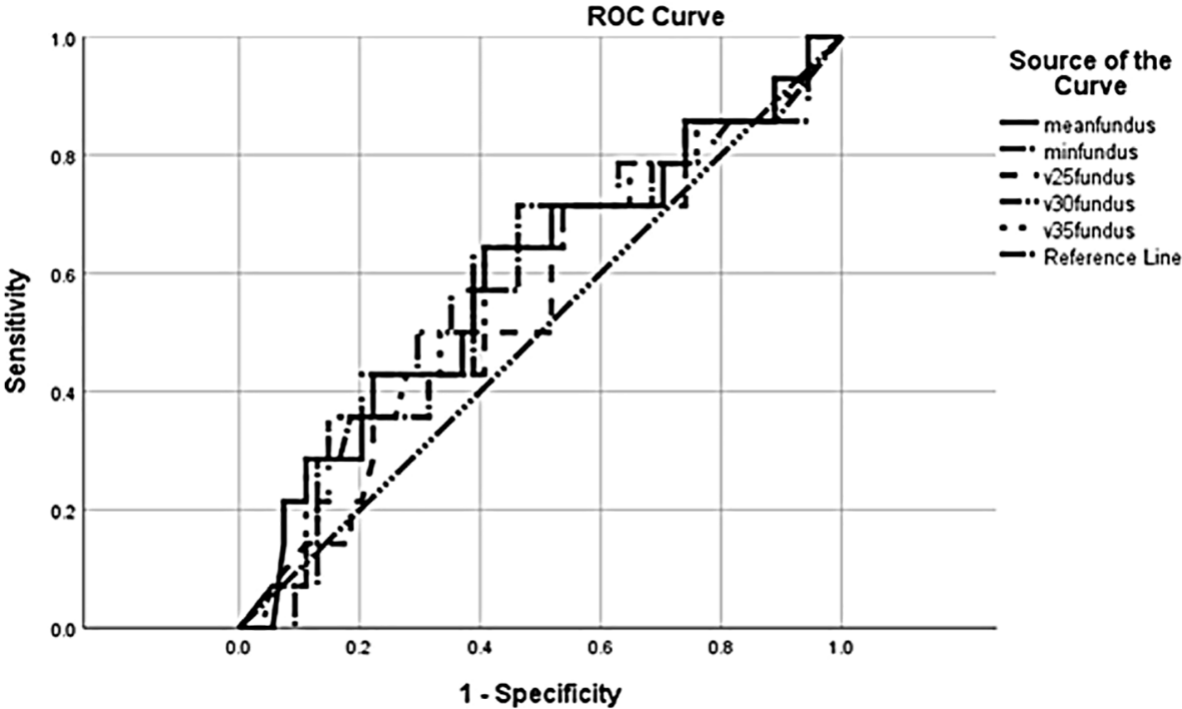
Receiver operating characteristic (ROC) curve for anastomotic leak in relation to radiation dose.

The mean fundus V25, V30, and V35 were all numerically higher in the anastomotic leak group (**Table 2**), though these differences did not reach statistical significance (*p* = 0.58, *p* = 0.39, and *p* = 0.30, respectively). Dose as a graduated variable was used to generate a receiver operating curve for the estimation of leakage risk and no specific cutoff point was identified to predict future leaks (**Figure 1**).

## Discussion

The precise impact of radiotherapy dose and specific recommendations regarding dose constraints as they relate to the risk of anastomotic leakage in the treatment of LAEC remain an area of uncertainty. Data from mostly retrospective reports to date have been widely variable and inconclusive.

In the above retrospective analysis of our prospectively collected relatively large and modern institutional experience, we did not identify a significant correlation between specific radiation dose parameters or delivery technique with the rate of postoperative anastomotic leak following nCRT. Mean V25, V30, and V35 to the region of the future anastomosis did not correlate with risk of anastomotic leakage. Our findings are consistent with other retrospective series, including two reports by Koeter et al., the first of which comprises 53 patients and the other with a larger retrospective report, which found no association between anastomotic leakage of nCRT and V20–V40 of the future anastomotic region (11, 16).

More broadly, two meta-analyses of neoadjuvant therapy and their associated complications showed no significant increase in postoperative morbidity and perioperative mortality with the addition of preoperative chemotherapy or chemoradiotherapy for resectable esophageal and GEJ cancer (4, 17). Similarly, the rate of anastomotic leakage was not significantly increased compared to those undergoing surgery alone. This was further supported in a large multicenter series comparing anastomotic leak rates between patients receiving nCRT compared to surgery alone, with an incidence of 8.8% *vs* 11.3%, *p* = 0.228 (5). A report from the Society of Thoracic Surgeons comprising data from 7,595 esophagectomies concluded that radiotherapy was not a predictor for leakage, with rates among those undergoing surgery alone *vs*. neoadjuvant radiation of 10.4% *vs*. 11.2%, respectively (18).

Other series, however, have found radiation dose to the gastric fundus to be an independent predictor of anastomotic complications among this patient cohort. In their review of 54 patients who received neoadjuvant 36 Gy with cisplatin and 5FU, Vande Walle et al. reported a 13% rate of early anastomotic complications, and a significant association with dose to 50% of the gastric fundus, recommending limiting the D50 to 29 Gy (10). Details of the precise radiation delivery method, however, were not described, and the gastric fundus volume was retrospectively contoured for dosimetric analysis. The same group later published a larger cohort comparing outcomes between those receiving a prescribed dose of 36 Gy *vs*. 41–50 Gy, which did not show a significant association between surgical outcomes or complication rates and dose escalation (19). Juloori et al. reported a positive association between anastomotic leaks and location of the anastomosis within the neoadjuvant radiation field, though a correlation was not drawn to a particular maximum or volumetric dose (9).

Further confounding the potential association of anastomotic leaks and specific aspects of nCRT is the influence of tumor location and surgical field. In a retrospective series of 51 patients, Bang et al. found an association between anastomotic complication after nCRT and a higher mean radiation dose to the esophagus at the level of the azygous vein and lower (20). Mean gastric dose, however, was not a significant factor predictive of anastomotic complications.

Finally, some have suggested that more conformal radiation delivery methods, such as IMRT, may have an impact on the risk of postoperative leakage. Wang et al. found a significant association between lower rates of postoperative GI complications and the use of IMRT compared to 3D-CRT (9.8% *vs*. 11.5%, *p* = 0.004) (21). Patients in this series were treated between 1998 and 2011 *vs*. our study cohort treated from 2016 to 2018, between which technologies for delivery of both 3D-CRT and IMRT as well as daily image guidance capabilities evolved, perhaps obscuring the subtle difference that had earlier been revealed.

Limitations of this study include its retrospective design, which is inherently unable to account for all potential factors that could influence outcomes. All esophagectomies in this analysis were performed in one high-volume surgical department with standardized procedures, though there may exist variation between surgeons. All radiation planning was performed by a single senior radiation oncologist, thereby limiting that confounder. Finally, varying individual anatomy including the spatial relationship between the esophagus and gastric fundus and configuration of vasculature relative to the relevant organs could influence differences postoperatively across patients, which is difficult to assess retrospectively.

In conclusion, our analysis of patients with locally advanced carcinoma of the esophagus and GEJ treated with nCRT demonstrated no significant association between specific radiation dose parameters or delivery technique and postoperative anastomotic leak. While data suggesting a specific dose constraint to the gastric fundus are inconclusive, attempting to limit excess radiation dose to the region of the lower esophagus and gastric fundus is a reasonable recommendation and may help mitigate the risk of this important postoperative complication, though at this time, definitive guidance for a specific constraint is lacking. Further investigation in well-controlled prospective studies to quantify the relationship between radiation and anastomotic leakage is warranted.

## Data availability statement

The raw data supporting the conclusions of this article will be made available by the authors, without undue reservation.

## Ethics statement

The studies involving human participants were reviewed and approved by Helsinki Committee of Beilinson Hospital, Rabin Medical Center. Written informed consent for participation was not required for this study in accordance with the national legislation and the institutional requirements.

## Author contributions

All authors contributed equally to this work. YK contributed to the study conception and design, data collection, analysis, interpretation of data, and drafting of the initial manuscript (first authorship). NK and OS were responsible for data collection. SG was responsible for contouring on the CT simulation slides. YN was responsible for the statistical analysis. HK contributed to the drafting of the initial manuscript. EyF contributed to manuscript review. BB contributed to study analysis, interpretation of data, and drafting of the initial manuscript. AM contributed to data collection and analysis. AP contributed to study analysis, interpretation of data, and drafting of the initial manuscript. ElF contributed to study analysis, interpretation of data, and drafting and editing of the final manuscript (last/senior authorship). All authors contributed to the article and approved the submitted version.
